# Vaccination with inactivated virus against low pathogenic avian influenza subtype H9N2 does not prevent virus transmission in chickens

**DOI:** 10.1016/j.jve.2021.100055

**Published:** 2021-09-22

**Authors:** Hongrui Cui, Mart CM de Jong, Nancy Beerens, Monique M. van Oers, Qiaoyang Teng, Luzhao Li, Xuesong Li, Qinfang Liu, Zejun Li

**Affiliations:** a Shanghai Veterinary Research Institute, Chinese Academy of Agriculture Sciences, Shanghai, China; b Quantitative Veterinary Epidemiology, Animal Sciences Group, Wageningen University & Research, the Netherlands; c Wageningen Bioveterinary Research, Wageningen University & Research, Lelystad, the Netherlands; d Laboratory of Virology, Plant Science Group, Wageningen University & Research, the Netherlands

**Keywords:** LPAI, H9N2, Transmission, Vaccination, SIR model, Chickens

## Abstract

H9N2 subtype avian influenza has spread dramatically in China ever since first reported in the 1990s. A national vaccination program for poultry was initiated in 1998. Field isolation data show that the widely used inactivated H9N2 vaccine does not provide effective control of the transmission of this low pathogenic avian influenza (LPAI) virus in poultry. Current research has focused on two reasons: (i) insufficient immune response triggered by the vaccination with the inactivated virus, (ii) the occurrence of escape mutants selected by vaccine-induced immune pressure. However, the lack of effectivity of the inactivated virus vaccine to sufficiently reduce transmission has been noticed. We mimicked the natural infection and transmission process of the H9N2 virus in vaccinated and non-vaccinated chickens. A statistical model was used to estimate the transmission rate parameters among vaccinated chickens, varying in serum hemagglutinin inhibition titers (HIT) and non-vaccinated chickens. We demonstrate, for the first time, that the transmission is not sufficiently reduced by the H9N2 vaccine, even when vaccinated chickens have an IgG serum titer (HIT>2^3^), which is considered protective for vaccination against homologous highly pathogenic avian influenza (HPAI) virus. Our study does, on the other hand, cast new light on virus transmission and immune escape of LPAI H9N2 virus in vaccinated chickens populations, and shows that new mitigation strategies against LPAI viruses in poultry are needed.

## 1 Introduction

Low pathogenic avian influenza (LPAI) viruses can still cause severe economic losses in the poultry industry,^1^ even though mortality is much lower than that of HPAI. The H9N2 subtype LPAI virus has attracted attention for its wide range spread in many host species, as well as for the fact that it has become endemic in commercial poultry in many areas, affecting poultry productivity. Besides being a threat to the poultry sector, the H9N2 virus also poses risks for public health as it can transmit from poultry to humans.^2^, ^3^, ^4^

Theoretically, infectious diseases can be controlled by a stamping-out policy or vaccination.^5^^,^^6^ Studies show that vaccination can reduce the transmission of HPAI H7N7^7^ and H5N1^8^ in chickens. Hence, a similar reduction in transmission was expected for LPAI viruses after vaccination. To control the spread of H9N2 viruses in China, inactivated virus vaccines in poultry were licensed and implemented in 1998.^9^ In reality, the poultry industry update privately inactivated vaccines based on their own virus strains noted from timely surveillance, instead of depending on the national commercial H9N2 vaccines in poultry. However, outbreaks of H9N2 viruses continue to be reported from vaccinated poultry farms.^10^ The failure of vaccination might be because of inefficient application, low dose, and low vaccination coverage (especially in the household sector).^11^^,^^12^ Moreover, the continuing transmission in combination with the intensive long-term usage of the inactivated virus vaccine may have led to antigenic changes leading to immune escape.^13^^,^^14^ Due to these possible failures of vaccination in poultry farms, vaccination was suggested as part of an overall integrated control strategy, including continued nation-wide surveillance, farm biosecurity, and DIVA (Differentiating Infected from Vaccinated Animals)) strategy.^6^^,^^15^

The inactivated virus vaccine was shown to be able to induce a strong immune response against the LPAI H9N2 virus in duck and chickens (using 2^8.5^ and 2^6^ EID_50_/0.1 ml of virus for antigen production, respectively), and showed significant reduce in virus shedding in the lab.^16^^,^^17^ Studies on virus shedding after vaccination also suggested a reduced shedding after vaccination with the H9N2 vaccine.^17^^,^^18^ However, direct information on the effect of vaccination to prevent transmission of H9N2 viruses was yet not available in these reports.

In this study, we quantified the transmission parameters of an H9N2 virus in chickens vaccinated with a homologous inactivated virus vaccine. Chickens after vaccination was used to study the effect on transmission following an optimized experimental design.^8^ The stochastic SIR model was used to estimate the transmission rate parameters among vaccinated chickens using a GLM (Generalized Linear Model) statistical approach.^19^ No significant reduction of the reproduction ratio (R) was observed in chickens after vaccination, indicating inactivated virus vaccine failed to stop the transmission of LPAI H9N2 virus in this study. The quantified transmission parameters are essential information for vaccine development and vaccination strategies to control LPAI in poultry.

## 2 Method and materials

### 2.1 Ethics statement

All animal experiments were executed according to the recommendations in the Guide for the Care and Use of Laboratory Animals of the Ministry of Science and Technology of the People's Republic of China. Animal welfare in the European Union^20^ was referred to ameliorate animal suffering. The Animal Care and Use Committee of Shanghai Veterinary Research Institute (SHVRI) reviewed the protocols including operative details of euthanasia via air inhalation (inhalational agent is carbon dioxide). The permit number was SHVRI-C-2019-0628-049. The chickens were regularly fed and maintained by technicians at the Research Team of the Etiologic Ecology of Animal Influenza and Avian Emerging Viral Disease (Research Team for short) at SHVRI. All experiments involving viruses were performed within the Biological Safety Level 2 facility at the Animal Centre of SHVRI.

### 2.2 Virus and animals

The LPAI H9N2 virus, A/chicken/Jiangsu/A2093/2011(KP866088.1, A2093), used to produce inactivated virus vaccine and viral inoculation in all the transmission experiments was provided by the SHVRI Research Team. This virus was propagated in 10-day-old specific pathogen-free (SPF) embryonated chicken eggs (ECEs) (Beijing Merial Vital Laboratory Animal Technology Co., Ltd). Allantoic fluid was harvested 48 h after inoculation and stored at −80 °C. The titer of the A2093 virus was determined and the 50% embryo infectious dose (EID_50_) was calculated using the Reed & Muench method.^21^^,^^22^

The White Leghorn chickens used in the experiments were hatched from SPF ECEs and raised in high containment facilities with an isolated air-circulation purification system. Enough water, proper food, and room for the chickens’ social interaction, foraging, and exercise were provided. Manure was removed at the end of the experiment.

### 2.3 Inactivation and vaccination

The A2093 virus (10^9^ EID_50_/0.1 ml) was inactivated by incubation of the virus stock with 1:2000 β-propiolactone for 12 h at 4 °C. The residual β-propiolactone was evaporated at 37 °C for 2 h. Complete inactivation was confirmed by two passages of inoculation in ECEs for at least 72 h. Each passage had to be negative in Hemagglutination (HA) assay as a criterion for successful inactivation. The live virus displayed 2^10^ HAU before inactivation and 2^6^ HAU after inactivation. The inactivated A2093 strain was then mixed with Montanide VG71(0.85 g/cm^3^) adjuvant at a volume ratio of 3:7 to guarantee good antigenicity. A total of 45 three-week-old SPF chickens were vaccinated with this virus emulsion (vaccine) by intramuscular injection in the legs. Twenty chickens were injected with 0.1 ml of the 2^6^ HAU/ml vaccine with the expectation of a low antibody response, and 25 were injected with 0.8 ml of the 2^6^ HAU/ml vaccine to achieve a high antibody response. Please note that chickens with a higher vaccine dose can still have a low antibody response.

### 2.4 Transmission experiments

Sera of vaccinated chickens were checked every day for two-weeks post-vaccination and collected until the antiserum level was stable. Hemagglutination Inhibition (HI) assays were carried out with eight HA units of live A2093 virus as antigen to determine the HI titers (HIT) of the sera. All assays were performed in duplicate. Based on their HITs, the vaccinated chickens were separated into the high HIT group (serum HIT higher than 2^3^) and low HIT group (HIT lower than or equal to 2^3^). This cut-off was based on the titers shown to be effective for HPAI viruses when vaccinated with low doses, which better reflects the field situation [19]. A control group was composed of unvaccinated SPF chickens. In each group, at least 10 chickens were selected based on the HIT level; half of the chickens received inoculation, the other half were recipients for transmission.

In Experiment 1, the inoculated chickens were infected with 10^7^ EID_50_ of the live A2093 virus at 0.1 ml intra-nasally and 0.1 ml intratracheally; in Experiment 2, 10^6^ EID_50_ viruses were used for inoculation in the same way. A 10-fold lower dose of the A2093 virus was applied in the second experiment to test whether the virus dose used to challenge the birds was too high to be able to see the transmission prevention of the vaccine. On day 1 post-inoculation (d.p.i.), the contact chickens from the same HI group were added to the containment unit in which the inoculated birds were kept. Chickens in the same group were free to contact with each other, and to share the food and water supply, and thus were able to have contact with others, for example via excrement.

Oropharyngeal and cloacal swabs of all animals were collected on d.p.i. 1, 3, 5, 7, 9, and 14, and were stocked in 1 ml phosphate-buffered saline (PBS) soon after collection. Each of the samples was mixed thoroughly and centrifuged at 12000 g for 10 min at 4 °C before the supernatants were collected and stored at −80 °C. On d.p.i 14, the chickens were euthanized, and the serum was collected for HI assays.

### 2.5 Virus quantification

In Experiment 1, the viral titers of the oropharyngeal and cloacal swabs were determined using the 10-day-old ECEs as previously described^23^ and calculated using the Reed & Muench method.^22^ The detection limit of the method was set at 0.98 log10-EID_50_/100 ul, when one of the three eggs incubated with the undiluted swab samples was positive in the HA assay.

In Experiment 2, samples were tested by two-step reverse transcription (RT) quantitative polymerase chain reaction (qPCR).^24^ Chicken embryo allantoic fluid containing 10^6^ EID_50_/100ul H9N2 virus was diluted into 10^5^, 10^4^, 10^3^, 10^2^, and 10 EID_50_/100 ul for the standard curve. Allantoic fluid from SPF ECEs was used as negative control. Viral RNA of all samples was extracted using QIAGEN Viral RNA Isolation Kit, and the cDNA was synthesized with Transcriptor High Fidelity cDNA Synthesis Kit (Roche) following the manufacturer's protocol. The qPCR specific to the M gene of the influenza virus was performed by using AceQ Universal U^+^ Probe Master Mix V2 (Vazyme) following the manufacturer's protocol. Threshold cycle (Ct) values of all the standard and swab samples and negative control were obtained. Standard curves were generated with the corresponding Ct values as its viral titers. Data were regarded as reliable only when the R-square value was above 0.996 in the data trendline. The Ct values were converted into logarithmic viral titers (log10-EID_50_/100ul) based on the standard curves and the corresponding regression line equations. The mean and 95% confidence interval (CI) of the swabs from SPF chicken were referred for a negative background control in the RT-qPCR method. The upper boundary of the 95% CI (1.97 log10EID50/100ul) was considered positive for virus shedding in this RT-qPCR method.

### 2.6 Rank test on virus shedding

For the total shedding value, we summed up the viral titers (log10-EID_50_/100ul) of all positive oropharyngeal samples of every chicken in the three Experiment 1 and 2 groups.

Firstly, we compared the total shedding amount of the inoculated and contact individuals in every group to test if the different inoculation doses in two experiments influenced the virus shedding for inoculated chickens compared to the contact ones. If inoculated chickens shed more than the contact infected, the inoculation dose could have been too high compared to the dose the contact chickens were exposed to. The datasets were filtered into high HIT, low HIT, and control groups, setting experiments (Exp1 and Exp 2) as the factor with two levels.

Secondly, virus shedding was also compared between the vaccinated chickens (High HIT group/low HIT group) and non-vaccinated chickens (Control group) to evaluate the effect of this vaccination on reducing virus shedding. Datasets were filtered into inoculated and contact, setting group as the factor with two levels (high HIT vs. control or low HIT vs. control, high HIT combined low HIT vs. control).

A Wilcoxon rank test^25^ was performed using RStudio^26^ (Script 1 in appendix Rscript.txt). The exact p-value was computed for the two-tailed test.

### 2.7 Data collection for SIR model

The in-contact or recipient animals at the start were counted as “susceptible” (S), as is the convention in the SIR model. Furthermore, based on virus quantification, inoculated and any contact animals infected in the course of the experiment were counted as “Infectious” (I) from the first day (Date of Starting, DS) they were found to be positive until the last day a positive sample was found (Date of Ending, DE). After the end of the excretion period, the animals were counted as recovered (R), in a total population with N individuals: N=S + I + R.

A “case” (C) was noted whenever a contact animal was infected, i.e., once an individual changed from the susceptible state (S) to the infected/infectious state (I), the population composition changed into (S-1, I+1). We defined the transmission rate parameter (β) for the transmission rate, βSI/N; and the recovery rate parameter (α) for the recovery rate, αI. We collected the transmission-related data (S, I) to estimate the transmission parameters, especially β in the SIR model. Only the individual chicken with viral titers of the oropharyngeal samples above each cutoff level was counted for statistical analysis. To obtain sufficient statistical power to detect differences between vaccinated and unvaccinated groups, we planned to start the experiment with I_0_ = S_0_ = 5 chickens in each group.

### 2.8 Estimation of transmission parameters

We used the reproduction ratio (R)^27^ to quantify the transmission, i.e. the average number of secondary cases caused by one typical infectious individual during its entire infectious period (T) in a fully susceptible population. The β was estimated with the number of infectious cases (C) in an interval, and the number of susceptible (S) and infectious cases (I) at the beginning of the interval. With the $=\frac{1}{T}$, we computed the reproduction ratio R = βT. The duration T of the virus excretion was estimated separately for vaccinated and unvaccinated chickens. The β was then estimated using a GLM (generalized linear model) implemented for our analysis in RStudio^19^ (Script 2 in appendix Rscript.txt).

The probability that a susceptible individual becomes infected during the observation time interval Δt, is thus given by:

(1) $$\text{p}=1-e^{-\beta \frac{I\Delta \text{t }}{N}}$$

In GLM analysis, a complementary loglog link function (ln [-ln(1-p)]) was used that transforms (1) into a linear relationship:

(2) $$\text{cloglog }\left(\text{p}\right)\;=\text{ ln }\left[-\text{ln }\left(1-\text{p}\right)\right]\;=\text{ ln }\left(\beta \right)\;+\text{ ln}\left(\frac{I\Delta \text{t }}{N}\right)$$

In this relationship, the dependent variable (p) is the number of cases (C) divided by the binomial total (S), and the offset equals ln $\left(\frac{I\Delta \text{t }}{N}\right)$. Furthermore, the error distribution is binomial. From the GLM analysis, we obtained the estimates of ln (β), its confidence intervals, and standard error.

To achieve a precise analysis of R from estimates of ln (β), ln(R) = ln (β) + ln(T), we counted the T value of every infected chicken in the contact population, of which we took the natural logarithm for the values of ln(T). Then the R and its confidence boundaries were estimated from the ln(R) and its confidence intervals. The variance of the estimator ln(R) was obtained using the following equation, assuming independence of ln(β) and ln(T):

Var[ln(R)]=Var[ln(β)]+Var[ln(T)]

The 95% confidence interval can be calculated as:

$$\bar{\ln\left(R\right)}\;\pm 1.96\sqrt{Var\left[\ln\left(R\right)\right]}$$

We also used the GLM analysis to find the effect of vaccination by separating the vaccinated chickens into high and low HIT groups with a threshold of 2^3^ HI units as previously estimated for HPAI.^8^ In this model, we estimated β_high_ for chickens having high HIT, β_low_ for chickens with low HIT, and β_control_ for non-vaccinated chickens. The dependent variable is the number of new cases C divided by S (C/S). Two dummy variables indicate either the high HIT group as 1 (0 otherwise) or the low HIT group as 1 (0 otherwise). As groups are homogeneous for the vaccination titer class, the regression coefficient c1 (see equation below) of the dummy variable for high titer gives the extra (or less) transmission in the high HIT group. So, this shows the combined effect of susceptibility and infectivity. The same is implied for c2, but then for the low HIT group. The equation for the model is:

$$\text{cloglog }\left(\text{p}\right)=\;{\text{c}}_{\text{0}}{+\text{c}}_{1}{\text{Ind}}_{\text{high}}\;{+\text{c}}_{2}{\text{Ind}}_{\text{low}}+\text{ ln}\left(\frac{I\Delta \text{t }}{N}\right)$$

Herein, ln(β) = c_0_+c_1_Ind_high_ + c_2_Ind_low._ Three βs can be obtained using the estimated regression coefficients from the GLM analysis:

$$\beta_{\text{control}}=e^{\text{c}0}$$

$$\beta_{\text{high}}=e^{\text{c}0+\text{c}1}$$

$$\beta_{\text{low}}=e^{\text{c}0+\text{c}2}$$

Estimation of transmission was performed on both experiments combined after testing for a possible difference between the two experiments.

### 2.9 Comparison of βs in the two experiments

With the two dummy variables set for the high and low HIT and control groups, we compared βs by using the *glm* function in RStudio. The Wald test for regression coefficients was applied to test whether the regression coefficient was different from 0. In the summary table of *glm,* function, z_values were generated with the corresponding probability (Pr (>|z|)) and used to evaluate the differences of the ln(β) values between the control group and the high HIT group (coefficient of Ind_high_) and or between the control group and the low HIT group (coefficient of Ind_low_). We used 95% confidence (i.e. 5% error rate) for difference analysis.

To measure the effect of the difference in the virus inoculum in the two experiments as separate factors, we set values 1 (Experiment 1) and 0 (Experiment 2) in the GLM model. We compared the datasets of the high and low HIT and the control groups from the two experiments.

### 2.10 Power calculation of the estimated R value

To draw a conclusion in case no significant difference of the transmission rate parameters between high HIT and control groups was found, we calculated the power of the test for biologically relevant differences. With the parameter βs from the vaccinated and control groups, we calculated the corresponding R_con_ (control group) and R_vac_ (vaccinated group). Practically, we expected a successful vaccination would reduce the R to below 1, meaning that the virus cannot persist in poultry. We took the null hypothesis as H_0_: R_vac_ = R_con;_ the alternative hypothesis was H_a_: R_vac_ < R_con._ The power calculation was based on the final size analysis of a corresponding pair-wise transmission experiment. It was shown to be a conservative estimate for the GLM analysis of any transmission experiment with any other group size containing the same number of S and I.^28^ The pair-wise final size difference can be analyzed as the difference between two binomial distributions (control vs. vaccinated group) with each having a different infection probability *p* for the contact animal in the pair to become infected. In this case, the p can be calculated using their Rs chosen under the null hypothesis (R_vac_ = R_con_ = 2.0) and under the alternative hypothesis (R_vac_<1 and R_con_ as estimated). By choosing R_vac_ = R_con_ = 2.0 under the null hypothesis, we know that we have the worst case for accepting the null hypothesis.^29^ This can intuitively be understood as then the probability for contact infection to occur is 0.5, which is the binomial distribution with the highest variance. Under the alternative hypothesis, infection probability is $p=\;\frac{R}{R+2\;}$ ^29^ With probability values of difference for every comparison, we calculated the cutoff value under H_0_ and the power under the alternative hypothesis.

## 3 Results

### 3.1 Vaccination with inactivated virus A2093-H9N2

**Fig. 1:**
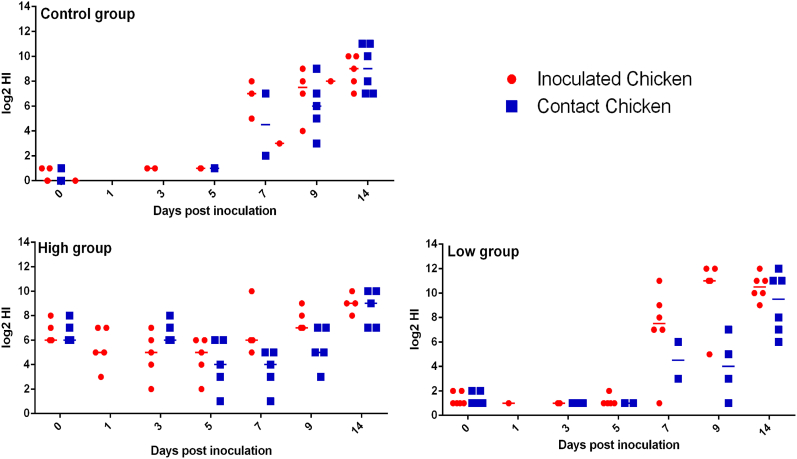
**HI titers in the sera of the chickens before and after inoculation in Experiment 1.** The high group and low group represent the high HIT group (serum HIT higher than 2^3^) and low HIT group (HIT lower than or equal to 2^3^), respectively. The Control group displayed the chickens without vaccination. All the HI titers of serum samples from individual chickens are displayed with median (bar) and range. Both the inoculated and contact chickens are shown.

**Fig. 2:**
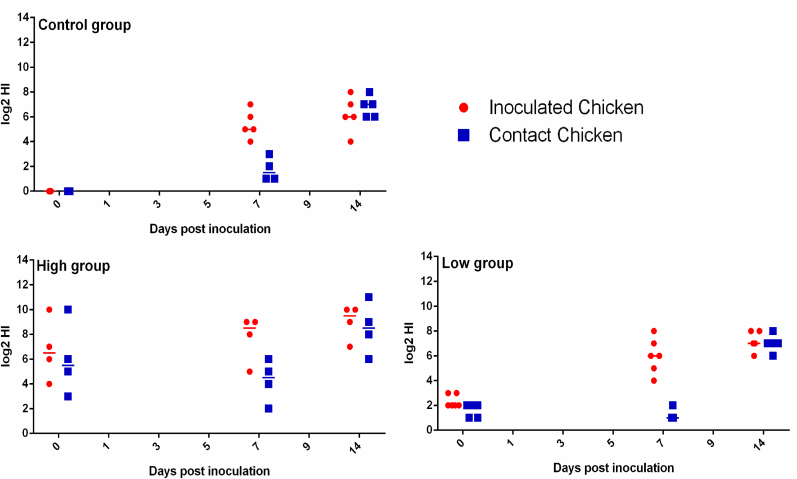
**HI titers in the sera of the chickens before and after inoculation in Experiment 2.** The high group and low group represent the high HIT group (serum HIT higher than 2^3^) and low HIT group (HIT lower than or equal to 2^3^), respectively. The Control group displayed the chickens without vaccination. All the HI titers of serum samples from individual chickens are displayed with median (bar) and range. Both the inoculated and contact chickens are shown. (in black and white).

The HIT of the serum from the vaccinated chickens was collected three weeks post-vaccination when the antibody levels were detected to be stable. The HIT of the chickens before inoculation was recorded at 0 d.p.i. (Fig. 1, Fig. 2). The chickens were grouped into high or low HIT groups based on the HI assay (Supplementary Table S1) as representative for a good or poor vaccination response. As in the field situation, the HIT varied between individuals after vaccination, but vaccination was considered successful for chickens obtaining HIT >2^1^. In Experiment 1, twelve chickens were included in the low HIT group, ten were included in the high HIT group, and twelve in the control group. In Experiment 2, only eight chickens obtained HI titers over 2^3^ after vaccination, twelve were included in the low HIT group, and ten in the control group. In each group, the number of inoculated and contact chickens was a 50:50 ratio.

### 3.2 Virus shedding

Antibody titers in the serum of both inoculated and contact chickens were checked in two experiments. All the contact chickens were infected according to a significantly increased HIT in serum on 14 d.p.i (Fig. 1, Fig. 2), indicating a successful infection with the virus. We observed no clinical signs in all the groups during the experiment. Only low levels of virus shedding were observed in cloacal swabs for this H9N2 strain in both experiments. Therefore, statistical analysis was performed on the data obtained from oropharyngeal swabs.

**Fig. 3:**
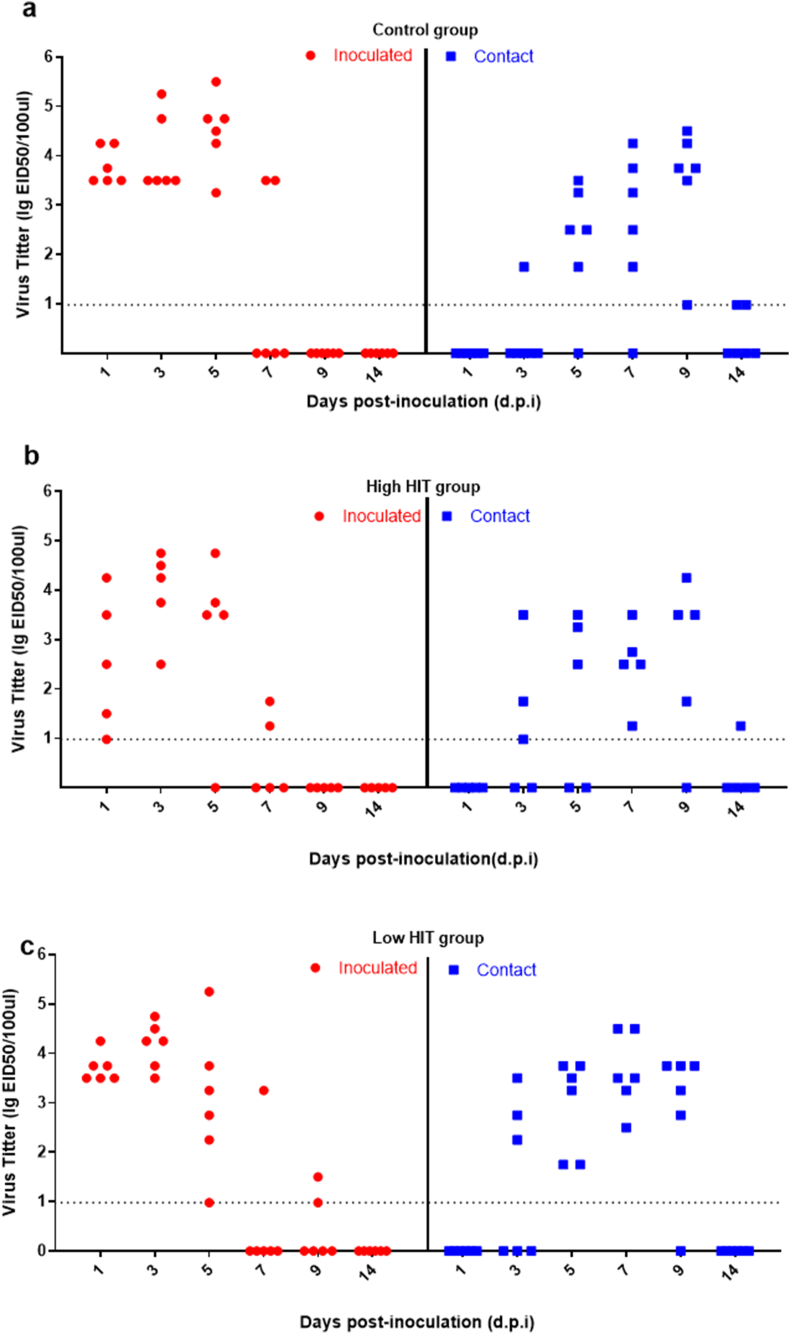
**Viral titers of the oropharyngeal swabs after inoculation in Experiment 1.** a, Viral titers of the oropharyngeal swabs in the control group; b, Viral titers of the oropharyngeal swabs in the high HIT group; c, Viral titers of the oropharyngeal swabs in the low HIT group. Viral titers were determined in ECEs and calculated using the Reed-Muench mathematical method. The background cut-off line is 0.98 lgEID_50_/100ul. All individuals which survived in this experiment and those with HI titers below the threshold are displayed on the x-axis. (in black and white).

In Experiment 1, all the inoculated chickens from three groups tested virus positive in the oropharyngeal swabs from 1 d.p.i. Contact chickens in the high HIT group were all tested positive at 7 d.p.i. (Fig. 3b); in the low HIT group (Fig. 3c), all contact chickens were positive at 5 d.p.i., similar to the control group, which had 5 of 6 individuals infected (Fig. 3a). All the details of the viral titers in the positive swabs are presented in Supplementary Table S2.

**Fig. 4:**
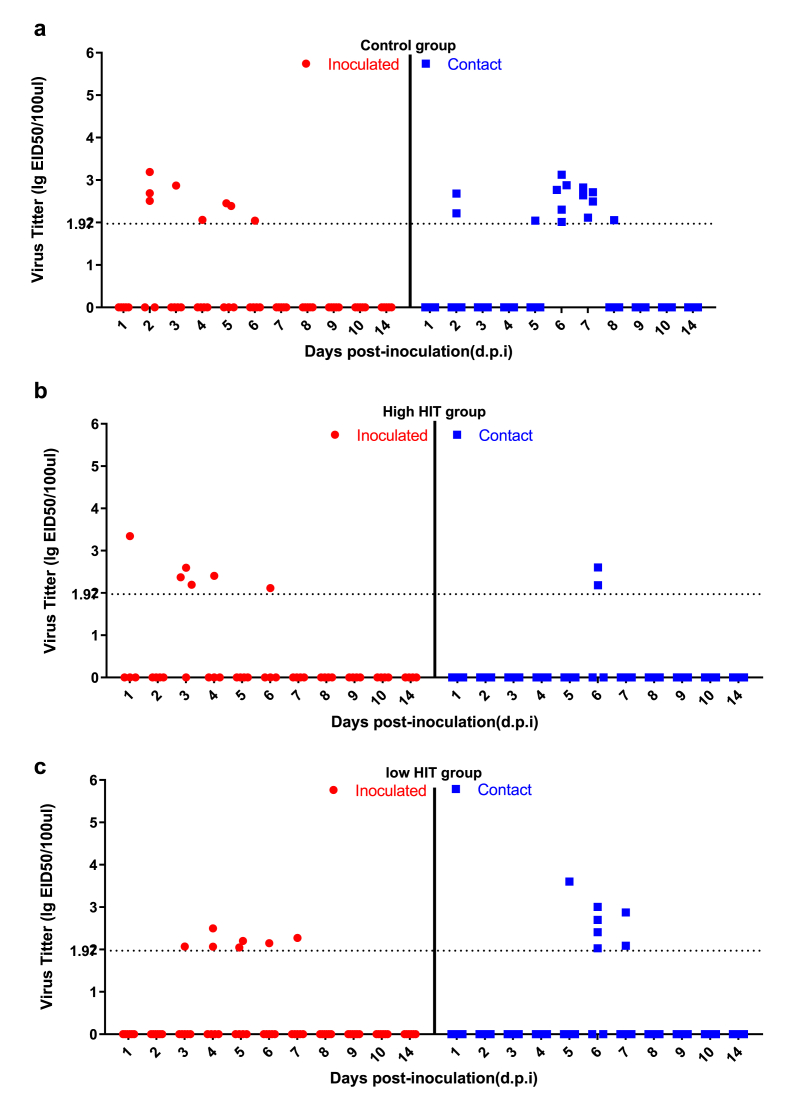
**Viral titers of the oropharyngeal swabs after inoculation in Experiment 2.** a, Viral titers of the oropharyngeal swabs in the control group; b, Viral titers of the oropharyngeal swabs in the high HIT group; c, Viral titers of the oropharyngeal swabs in the low HIT group. Viral titers were determined using qPCR and calculated into EID_50_ values based on the standard curve. The background cutoff line is 1.97 lgEID_50_/100ul. All the individuals were alive, but only those with viral titers above the cut-off are shown in the figure. (in black and white).

Based on the detection method, the cut-off value of 1.97 log10-EID_50_/100ul was set to count infectious cases and estimate the duration of virus shedding. The individuals with virus shedding below the cut-off were set as 0 (Fig. 4). Not all the inoculated chickens were infected after 24 h in each group, and they also had variable individual viral titers. In the control group, virus shedding was detectable in all of the contact chickens after 6 d.p.i. (Fig. 4a). In the high HIT group, only one had a titer above the cutoff line after 24 h (Fig. 4b). On 3 d.p.i., three of the inoculated chickens showed significant virus shedding and two contact chickens became infected on 5 d.p.i. In the low HIT group (Fig. 4c), two inoculated chickens shed viruses on 4 and 5 d.p.i and four of six contact chickens became infected on 6 d.p.i. Details in Supplementary Table S3.

### 3.3 Comparisons of virus shedding in rank test

For the analysis of the transmission, we assumed that contact infected and inoculated chickens in the different groups had the same infectivity. However, the infectivity of inoculation may depend on the inoculation dose. An indication for too high inoculation dose (e.g. 10^7^ EID_50_ in Experiment 1) would be when inoculated individuals shed more than the recipient contact individuals. Therefore, we compared the virus-shedding levels between the inoculated and contact individuals in the different groups in two experiments. The null hypothesis was that inoculated and contact chickens have the same virus-shedding level. In the Wilcoxon rank test (Table 1), p-values for the comparison between inoculated and contact chickens in Experiment 1 indicated the same distribution of the inoculated and contact population in the vaccinated population (p = 0.35 in high HIT group, p = 0.75 in low HIT group), but a biased shedding level in the control group (p = 0.0065). Comparing Experiment 1 with Experiment 2 showed that in experiment 1, virus shedding was significantly higher than in Experiment 2.

**Table 1:** Wilcoxon rank test of virus excretion comparing inoculated versus contact and Experiment 1 (Exp1) versus Experiment 2 (Exp2).

Comparisons	Datasets	Exp 1	Exp 2
**Inoculated vs. Contact**	High HIT Group	W = 8, p-value = 0.35	W = 0, p-value = 0.050
	Low HIT Group	W = 16, p-value = 0.75	W = 10, p-value = 0.56
	Control Group	W = 1, p-value = 0.0065^#^	W = 19, p-value = 0.17
**Exp1 vs. Exp2**^a^	High HIT Group	W = 58, p-value = 0.0024^#^	
	Low HIT Group	W = 95, p-value = 0.00029^#^	
	Control Group	W = 96, p-value = 0.018^#^	

a Sum of virus shedding of inoculated and contact chickens. # P-value <0.05 indicated significant difference.

Considering the influence of inoculation doses, a comparison of virus shedding from vaccinated versus non-vaccinated chickens was carried out separately for the two experiments. Table 2 shows that the virus shedding of the chickens in the vaccination group with relatively high HIT (>2^3^) was reduced compared to the non-vaccinated group in some comparisons: i.e. for experiment 1, this was in the inoculated group and experiment 2 in the contact infected group. In detail, in Experiment 1, six of the ten vaccinated chickens (high HIT group) obtained antisera titer at 2^6^ HIT, and four were above 2^6^ HIT, but the virus shedding level was not significantly different from the un-vaccinated group for both inoculated (p = 0.10) and contact chickens (p = 0.78). With less virus dose for inoculation in Experiment 2, virus shedding of chickens in the vaccinated group (high HIT group with five of eight chickens achieving more than 2^6^ HIT) was significantly less in contact chickens, at only (p = 0.025). Moreover, combining the data of high HIT and low HIT group as a vaccinated population (mixed individuals with good and bad antibody responses), the virus shedding was also reduced with a significant difference in inoculated chickens but not in contact chickens (W = 53, p-value = 0.044) from Experiment 1. However, in Experiment 2 vaccination significantly reduced the virus shedding in contact chickens (p-value = 0.042) compared to inoculated ones. All these biases could be due to the influences from different inoculation doses. After combining data from the two experiments (“Experiments combined” in Table 2), no significant difference was found with the Wilcoxon rank test for both contact and inoculated chickens.

**Table 2:** Wilcoxon rank test of virus excretion comparing the vaccinated (High HIT and low HIT group) versus non-vaccinated (Control group) chickens.

Comparisons	Datasets	Exp 1	Exp 2	Combined Experiments
**High HIT vs. Non-vaccinated**	Inoculated	W = 24, p-value = 0.10	W = 6, p-value = 0.65	W = 51, p-value = 0.56
	Contact	W = 16.5, p-value = 0.78	W = 15, p-value = 0.025^#^	W = 59.5, p-value = 0.20
**Low HIT vs. Non-vaccinated**	Inoculated	W = 29, p-value = 0.078	W = 14, p-value = 0.33	W = 67, p-value = 0.40
	Contact	W = 10, p-value = 0.20	W = 15, p-value = 0.22	W = 58, p-value = 0.83
**Vaccinated vs. Non-vaccinated**^a^	Inoculated	W = 53, p-value = 0.044^#^	W = 20, p-value = 0.68	W = 118, p-value = 0.39
	Contact	W = 26.5, p-value = 0.51	W = 30, p-value = 0.042^#^	W = 117.5, p-value = 0.41

a The vaccinated data was the sum of virus shedding from the High HIT and low HIT group; Non-vaccinated data was from the control group. # P-value <0.05 indicated significant difference.

### 3.4 Estimation of transmission rate parameters

The data of the transmission experiments used to estimate transmission parameters were observed as S, I, C, and N as described in the M&M above. The results are given in Table 3 and the result of the analysis is described below.

**Table 3:** Data abstracted from the transmission experiment for parameter estimation for the stochastic transmission model.

Experiment	Group	DS^a^	DE^b^	S	I	C	N
Exp 1	Control	1	3	6	6	1	12
	Control	3	5	5	7	4	12
	Control	5	7	1	11	1	12
	High	1	3	5	5	3	10
	High	3	5	2	8	1	10
	High	5	7	1	8	1	10
	Low	1	3	6	6	3	12
	Low	3	5	3	9	3	12
Exp 2	Control	2	3	3	5	0	10
	Control	3	4	3	3	0	10
	Control	4	5	3	4	1	10
	Control	5	6	2	5	2	10
	High	1	2	4	1	0	8
	High	2	3	4	1	0	8
	High	3	4	4	3	0	8
	High	4	5	4	2	0	8
	High	5	6	4	1	2	8
	High	6	7	2	3	0	8
	Low	3	4	6	1	0	12
	Low	4	5	6	3	1	12
	Low	5	6	5	3	3	12
	Low	6	7	2	5	0	12
	Low	7	8	2	3	0	12

Date of Start, the date when infection case(s) is observed; b, Date of Ending, the date when the infection case ends.

Supplementary Table S4 displays the basic datasets of the two experiments for estimation and pairwise comparison between groups in RStudio. The average duration of the observed shedding (T), the estimated βs, and the corresponding R values of the different groups in both experiments are listed in Table 4. In experiment 1, the average infectious periods were 7.6, 7.8, 6.5 in the high, low HIT, and control groups, respectively. Whereas, in Experiment 2, the average T of both high and low HIT groups dropped (2 for the high HIT group and 2.8 for the low HIT group) compared to that of the control group (T = 5.0 days).

**Table 4:** Three transmission rate parameters of three groups in different datasets from two experiments.

Experiments	Group	Coefficient	β^a^ (day^-1^)	T (days)	R^b^	0.95 CI of R	
						down	up
**Exp 1**	High HIT Group	−0.209	0.811	7.6	6.090	2.276	16.295
	Low HIT Group	0.007	1.007	7.8	7.727	3.007	19.860
	Control Group	−0.449	0.638	6.5	4.857	2.079	11.349
**Exp 2**	High HIT Group	−0.838	0.433	2.0	0.865	0.212	3.536
	Low HIT Group	−0.016	0.985	2.8	2.591	0.790	8.496
	Control Group	−0.251	0.778	5.0	3.562	0.833	15.227
**Exp 1&2**	High HIT Group	−0.442	0.643	6.0	3.305	0.740	14.766
	Low HIT Group	−0.003	0.997	5.8	4.988	1.290	19.284
	Control Group	−0.385	0.680	6.5	4.110	1.458	11.586
	Control&Low Group	−0.203	0.816	6.1	4.466	1.490	13.386

a β is calculated from exp (Coefficient).

b R is calculated from exp(lnR), lnR = lnβ+lnT.

In the pairwise comparison of the β between groups in the two separate experiments, no significant differences between the high HIT and control groups were found, nor between the low HIT and control groups (Table 5). Combining the data for the control and low HIT group (C&L), gave a combined estimated β_C&L_ for unvaccinated/poor-vaccinated chickens in each experiment. Even then, no significant differences were found comparing the combined group to that of well-vaccinated (high HIT group) chickens in either experiment (Table 5, p = 0.931 in Experiment 1, p = 0.379 in Experiment 2).

**Table 5:** Comparison analysis of lnβ between groups.

Experiments	Comparisons of lnβ	p value	Effect (lnβ)
Exp1	Control vs. High Group	0.702	0.240132
	Control vs. Low Group	0.451	0.456519
	Low Group vs. High Group	0.735	0.216387
	High Group vs. (C&L)	0.931	0.041886
Exp 2	Control vs. High Group	0.524	−0.5867
	Control vs. Low Group	0.758	0.2358
	Low Group vs. High Group	0.348	−0.8225
	High Group vs. (C&L)	0.379	−0.715
Exp 1&2	Control vs. High Group	0.911	−0.05693
	Control vs. Low Group	0.415	0.3828
	Low Group vs. High Group	0.594	−0.2389
	High Group vs. (C&L)	0.594	−0.2389
High HIT Group	Exp 1 vs. Exp 2	0.464	0.629
Low HIT Group	Exp 1 vs. Exp 2	0.972	0.02293
Control Group	Exp 1 vs. Exp 2	0.78	−0.1978
High Group vs. (C&L)	Trial I vs. Trial II	0.747	0.17664

We then conducted difference analysis between the two experiments separately for paired high, low HIT, and control groups, respectively. No significant differences were found in transmission parameters between paired HI level groups from the two experiments (Table 5). Practically, we combined the paired data of both experiments to estimate the transmission parameters without considering the inoculation dose. With the combined basic dataset (Table S4), we obtained more precise estimations for the transmission parameters of high, low HIT, and control groups, β_con_ = 0.680 day^-1^, β_high_ = 0.643 day^-1^, and β_low_ = 0.997 day^-1^. As in Table 4, the estimated T for the high HIT group was T_high_ = 6.0 days, R_high_ = 3.31 (95% CI, 0.740–14.766), for the low HIT group was T_low_ = 5.8 days, R_low_ = 4.99 (95% CI, 1.290–19.284); and for the control group it was T_con_ = 6.46 days, R_con_ = 4.11 (95% CI, 1.458–11.586). Pairwise comparisons were analyzed between the high HIT and control group using the same method, but again no significant differences were observed (p-value = 0.911); we also found no significant difference between the low HIT and control group (p-value = 0.415) (Table 5). We then combined the statistics of the control and low HIT groups (C&L), obtaining the combined estimated β_C&L_ = 0.82 day^-1^, and R = 4.47 for control combined with the low HIT group (95% CI, 1.490–13.386). Still, we found no significant differences between vaccinated and unvaccinated chickens (p-value = 0.594).

### 3.5 Power calculation

For the comparison of the R values between the vaccinated group with high titer and the unvaccinated control, we compared (S, I) = (6,6) for control to (S, I) = (5,5) for the high HIT group in Experiment 1; and (S, I) = (5,5) for control and (S, I) = (4,4) for the high vaccinated group in Experiment 2. In terms of power, this is the same as comparing between 9 pairs of (S, I) = (1,1) for the vaccinated high titer group and 11 pairs of (S, I) = (1,1) for the control group.^29^ The experimental data of the control group gave an estimated R_C_ = 4.1 with the number of sample pairs, N _C_ = 11. With the sample size of 9 pairs for the high HIT population and 11 pairs for the control, we obtained a p_value < 0.05 (0.04965) rejecting the null hypothesis (R_vac_ = R_con_ = 2). Then, under the alternative hypothesis with R_con_ = 4.1 and expected R_vac_ = 0.6, we obtained the power = 0.6505.

After having found no significant differences between the low HIT and control groups in the transmission parameter, we combined the data from the low HIT and control groups for precise estimation of the R parameter and larger sample size for higher power value. The experimental data of the unvaccinated population had a combined estimation β_C&L_ = 0.82 day^-1^, and an average T of 6.1 days, with R_C&L_ = 4.47. The number of the sample pairs in this poorly or non-vaccinated population was N _C&L_ = 23, and an estimated power of 0.7809 was obtained with p_value as 0.04866.

Therefore, we had more than 0.78 of power i.e. if vaccination after achieving HIT>2^3^ in the high HIT experimental groups would have stopped the transmission of LPAI H9N2 virus (R < 1), we would have detected it.

## 4 Discussion

In this study, we investigated the effect of the inactive-virus vaccine against LPAI H9N2 on the transmission of the homologous virus in chickens by combining the transmission experiments and SIR model for reproduction ratio (R). The starting assumption was that the transmission could successfully be stopped (R < 1) in a vaccinated chicken population if there is no antigenic difference between virus and vaccine. The stochastic SIR model was used to estimate transmission parameters from experimental data.^30^ In this study, we estimated the transmission rate parameter based on the H9N2 virus from oropharyngeal shedding, given that it is difficult to evaluate clinical protection for LPAI viruses via observing clinical signs from infected chickens.^31^^,^^32^

In the transmission experiments, virus shedding was detected and used to determine the infectious status of the chickens for both inoculation and contact (susceptible individuals). The SIR model does not include an exact value for virus shedding; the infected case is defined based on virus detection of either 0 (negative) or >0 (positive). Therefore, the viral titers determined using the 10-day-old ECEs with Reed & Muench method (Experiment 1), and by RT-qPCR (Experiment 2) were referred based on the methodology thresholds respectively. When challenged with 10^7^ EID_50_ of virus (Experiment 1), the transmission of LPAI H9N2 did not result in an R value below 1 in well-vaccinated chickens. With this high viral dose, all the inoculated chickens from the vaccinated groups were shedding virus via the oropharyngeal pathway, and total shedding levels between inoculated and contacts were not significantly different according to the Wilcoxon rank test. However, the high virus dose contributed to the high shedding of inoculated chickens in the control group (Table 1). We performed another independent transmission experiment with 10^6^ EID_50_ viruses to avoid the bias of data from a single high inoculation dose. In this study, where we simulated the natural infection route via contact, virus shedding was still observed for both inoculated and contact chickens. No significant difference in the total shedding amount was found between inoculated and contact chickens in the Wilcoxon rank test, based on the combined data in two experiments. For the effect of this vaccine on the virus shedding, reduction in oropharyngeal virus shedding of contact chickens from the high HIT group was observed compared to that from the control group when the challenging dose was 10^6^ EID_50_. However, when combined with the data from two experiments, there was no significant difference between vaccinated (both in high HIT and low HIT group) and non-vaccinated chickens in the Wilcoxon rank test. Vaccination with inactivated virus vaccine did provide certain protection for those individuals obtaining high antibody response, but this protection did not stop virus shedding completely. This result may be biased by the fact that we have limited data for this group, and the infectious period was very short. The effects of the virus shedding on transmission were further analyzed using transmission models and statistical analysis.

In the statistical analysis, the inoculated dose was included as a modifying factor to obtain an overall estimation of the vaccination effect. Even though when challenging with less virus dose there were fewer infectious individuals, a shorter infectious period and lower total shedding amount were observed, still, the infection of contact chickens was observed, showing highly increased HI titers at the end of the experimental period (Table S1, except for the bird NO. 33). This suggests that vaccinated chickens can still be infected by the 10^6^ EID_50_ viruses, and the transmission rate is reduced by a high level of antibody. But to achieve an optimal transmission model reflecting the transmission process with different viral doses and shedding level, as occurs in the field, we combined the two independent experiments together for analysis of the vaccination factor solely so that it would give a better estimation of the transmission rate parameters. The combined datasets showed that both virus shedding level and transmission rate were not significantly reduced by vaccination.

We applied the GLM to analyze our transmission experiments, as this is feasible for a heterogeneous population, providing higher power to find a difference in transmission between two treatment groups.^29^ After observing no differences between the control and low HIT group, we combined the parameters of these two groups (C&L). We also found no significant differences between the high HIT group and the combined (C&L) (p = 0.594). We thus accepted the null hypothesis that transmission rate parameters of vaccinated and unvaccinated chicken were not significantly different. As we only had a few groups and no large ones, we did a power analysis to evaluate the confidence of the conclusion drawn from this sample size. With an expected R value of 0.6 as an indication for successful vaccination, the power in our experiment was 78%, which is somewhat less than 80% due to the uneven sample sizes of the vaccinated and unvaccinated groups, and the lower number of individuals in the high HIT group. However, our results strongly suggest that even successful vaccination (in terms of achieving high titers > 2^3^) cannot stop the transmission of H9N2 among vaccinated chickens.

In contrast to our results for H9N2, previous research showed a significant reduction in the transmission of some H5 and H7 HPAI subtype avian influenza in chickens after vaccination.^7^^,^^33^ In addition, research by van der Goot et al.,^34^ showed a considerable reduction in transmission for H5N1. H9N2 vaccination was found to provide protective efficacy against disease for H9N2 avian influenza virus in ducks after intravenous injection of the virus.^16^ However, in those vaccination and challenge experiments, the virus could still be detected (at low levels) in nasal or cloacal swabs during the experiments.^35^, ^36^, ^37^ For example, in the study suggesting that inactivated H9N2 vaccines containing at least 250 HAU/dose will minimize virus shedding in SPF chickens,^38^ in 20%–50% chickens, 250 HAU/dose vaccination was detected with 2.3–2.48 EID_50_/ml virus shedding. However, the transmission resulting from this virus shedding was not subsequently estimated. For the vaccines against HPAI in poultry, good protection against clinical signs and mortality was achieved,^39^ whereas another study identified that a single vaccination dose hardly reduced transmission of the H5N1 virus in ducks, one week after vaccination.^40^ Because no clinical signs and mortality were observed in chickens infected with the LPAI H9N2 virus, the individual protection of this vaccine was evaluated based on virus shedding. In our experiment, the inactivated H9N2 vaccine did induce an immune response, as we measured an increase of antibodies in the serum. With reduced virus shedding, the transmission still continued in the population. Further research is required to provide more insights into the immune response and the relationship between vaccination and transmission of LPAI viruses.

## 5 Conclusion

In our research combining an experimental transmission study with mathematical modeling, we show that inactivated H9N2 vaccine is able to provide individual protection, but unable to stop the transmission of the H9N2 virus in chickens. The power of the statistical test used was 78%. Transmission modeling can give a statistical estimation of the vaccine effects on virus transmission in certain populations, thereby providing guidance for control strategies.

## CRediT author statement

Hongrui Cui: Conceptualization, Formal analysis, Visualization, Investigation, Writing - Original Draft, Writing - Review & Editing.

Mart CM de Jong: Conceptualization, Methodology, Formal analysis, Supervision, Project administration, Writing - Review & Editing.

Nancy Beerens: Writing - Review & Editing, Validation, Visualization.

Monique M. van Oers: Writing - Review & Editing, Validation, Visualization.

Qiaoyang Teng: Resources.

Luzhao Li: Investigation.

Xuesong Li: Resources, Investigation.

Qinfang Liu: Resources, Investigation.

Zejun Li: Conceptualization, Writing - Review & Editing, Supervision, Funding acquisition.

## Funding

This work was supported by the 10.13039/501100012166National Key Research and Development Program of China (2016YFD0500204) and the 10.13039/501100005196Chinese Academy of Agricultural Sciences of Technology Innovation Project.

## Declaration of competing interest

The authors declare that they have no known competing financial interests or personal relationships that could have appeared to influence the work reported in this paper.
